# American Medical Association

**Published:** 1855-06

**Authors:** 


### AMERICAN MEDICAL ASSOCIATION.

TUESDAY, MAY 1st, 1855.

The session was opened at 11½ A.M., by the President, Dr. C. A. POPE, of St. Louis, taking the Chair; Drs. N. S. DAVIS, of Illinois, and John GREEN, of Massachusetts, Vice Presidents, occupied seats on either side; Secretaries, Dr. FRANCIS WEST, of Philadelphia, and Dr. E. S. LEMOINE, of St. Louis.

The President called the Association to order, and announced that the first order of business was the reception of the report of the Committee of Arrangement on the Credentials of Members.

Dr. Hays, Chairman of that Committee, before presenting the report, begged leave on behalf of the medical profession of Philadelphia, to greet the members with a sincere and cordial welcome, and to assure them that every endeavor had been made to affect such arrangements as would facilitate the deliberations of the Association, promote the comforts of its members, and render their sojourn in Philadelphia agreeable. This, he stated, was a duty incumbent on the profession of this city and equally a gratification to them to execute. "Eight years," he said "have elapsed since the Association was here organized, and our brethren in every city in which the Association has since met, have received our Delegates with a generous and munificent hospitality which we dare not aspire to rival, and can scarcely hope to approximate. Should our effort then fall short of fulfilling our wishes, I must beg you to, at least, accept the assurance that it is our earnest desire to do everything in our power to demonstrate our profound respect for the assembled wisdom of the noblest of Professions."

Dr. Hays reported that 337 Delegates had already registered, and stated that the Committee of Arrangements were now in session, and busily engaged in the performance of their duties.

As the next order of business was the calling of the roll, Dr. H. hoped that the reading of the report would be dispensed with as unnecessary, which was acceded to.

The roll was then called by the Secretary, Dr. West, 337 Delegates answering to their names.

The President, Dr. Chas. A. Pope, being, on motion, invited to address the Association, spoke as follows:

[We omit the first part of the address.]

The object of this Association is to do something to advance the profession towards the far-distant goal of perfection—to aid the solution of some of the problems and enigmas of life and organization—to add some material to the growing temple, whose foundations were so firmly laid by the Coan sage—and to do its part, as best it may, in the cause of humanity. Nor do I think that, so far, it has altogether failed. Many valuable contributions to science have been elicited—professional ambition has been stimulated, an *espirit-de-corps* has been successfully evoked and established. The strength of the profession has acquired additional power by the union of its members. This association has been to physicians what the railroad and electric wires are to commerce, and the interchange of useful knowledge to States and nations. It has made us one, and, as I have just remarked, in unity there is power.

This association has stimulated thought. Chaotic and void would forever remain the masses of facts, accumulated by the observations of ages, but for the co-ordinating and logical power of reason. It sits in judgment on the silent phenomena, as a "refiner of fire, and a purifier of silver." It forces the voiceless facts to mount the tripod of the oracle, and speak forth words of wisdom. The scalpel, the crucible, the microscope, may be subsidiary to its purposes and ends, but they cannot supply its place. Fixed and patient thought in medicine, as in the other departments of science, is the Aladdin's lamp that lights the footsteps of the discoverer. To stimulate attention and thought, is to accelerate many a new discovery, to hasten the advent and establishment of important principles yet in the womb of the future. May not our Association do this more effectually than it has hitherto done?

Let all the contributions be read and attentively considered. Such a course would certainly be more encouraging, as well as more respectful, to their authors. Let the reports be deliberately and fully discussed, and let them go forth to the world with the sanction or the criticisms of the Association. This would require time, it is true, but if we have time to meet at all, surely a few days would make but little difference. The good that would be effected would yield a ten-fold compensation for the time employed. Every one must admit that three or four days is too short a time for the Association rightly to fulfil its annual mission.

I would, moreover, respectfully suggest that time be taken for the discussion of some of the leading topics of medical philosophy. Amongst these, may be mentioned the nature, causes and treatment of cholera, yellow fever, et cetera—Hygiene, and the laws of health affecting masses of men—Quarantine—the causes of mortality among children—the chemical and vital doctrines of life. Questions like these, indicated a year in advance for discussion, would excite a carefulness of investigation, and a degree of attention and thought, which could not fail to clear away much of the darkness and doubt in which they are yet shrouded. Nothing so sharpens the intellectual powers as public debate. It fixes attention, and strains to the utmost every faculty. I have no hesitation in saying that facts enough have been accumulated to establish great and general principles, of which the medical world is yet in ignorance or doubt. Nothing would contribute more to demonstrate these principles than the collision of matured intellects in public debate. What a mass of facts and arguments and demonstration would be brought to bear on any of the subjects alluded to, if some of the best minds in the profession were to debate them, after a year's preparation! Observed facts are the crude materials of science—the intellect is the master builder of its august temple.

I make these suggestions for your consideration. All the scientific meetings in this country and in Europe, employ more time than ours has hitherto employed. Evidently we must protract our sessions, if we would render them as serviceable to science as they may be. No member of the Association will be required to remain longer than suits his wishes or convenience. Some fifty or sixty, more or less, would always be found to listen with eagerness to scientific papers, and engage with pleasure in scientific discussions.

The time has probably arrived for a change in our plan of organization, which will admit of the selection of a permanent place for the future meetings of the Association. There are evident advantages incident to both the migratory and stationary plans. These might, perhaps, be easily reconciled and secured. A proposition, if I mistake not, was made some years ago, by the Smithsonian Institution, and I would respectfully suggest, whether it would not be in accordance with the best interests of the Association, to hold biennial meetings in Washington, and the alternate ones, as now, at different points of our common country. We might thus secure all the advantages of a fixed abode, in the way of preserving the archives, making collections, etc., whilst by meeting in various localities, we could not fail to excite that wide-spread interest among the profession, and obtain such accessions of new members as would greatly enhance the high and useful objects of our Association. Should this proposal meet with your approbation, I would further intimate, that policy would perhaps require the meetings of the Association at the National Capital, to be held in the years of the short sessions of Congress.

I shall say but little of the legislative duties of the Association. I shall say nothing of the propriety or impropriety of getting laws passed to regulate the practice of medicine, and furnish standards for candidates for the doctorate. Perhaps the Association can do but little in this respect. Ours is a popular government, and the people are disposed to allow the largest freedom in everything pertaining to medicine, medical schools and physicians. Laws passed against quackery one year are revoked the next. Our country is the paradise of quacks. All good things have their attendant evils, and this unbridled liberty is one of the evils of a popular government. May we not hope, however, that even this evil may disappear, as general education and the cultivation of the masses advance? At any rate, the people are not yet dispossd to put down the quacks, nor to require too high a degree of qualification for those of the regular profession. After all, laws can make only mediocre physicians. They can require the candidates to know only so much—to be qualified to a certain degree; and this degree will always be far lower than that to which the true lovers of knowledge would attain, without any legislation on the subject. The greater lights of the profession cannot be manufactured after any process of legislative enactment. Thirst of knowledge, self love, philanthropy, burning ambition—these make the great physician and surgeon. These have made all the worthies of the past—not legislation. Legislation cannot drive the drone to the proud heights of professional eminence. When these heights are reached, it will be seen that the successful aspirant has been stimulated by a stronger power.

To him the laurel blossoms of renown and the life-giving mission of his art, are dearer and more attractive than was the mystic bough of the sibyl to the eager Æneas, or than the golden apples, guarded by sleepless dragons, to the Hesperian daughters.

What ever course you may think proper to pursue, I am sure that your objects will be, the advancement of science—the good of mankind—the honor and glory of the profession. We have the dignity and character of a noble calling to sustain—of a profession which has numbered, for two thousand years and more, some of the wisest and best men in all countries and all times. It is no trivial matter to sustain the rank and respectability of a vocation which can boast of a Hippocrates—a Harvey—a Hunter—of the most erudite and beneficent of sages and philanthropists the world ever saw—of a profession which has furnished to every nation its *clarum et venerabile nomen.*

On the eve of the battle of the pyramids, Napoleon exclaimed—"Soldiers! from the height of yon monuments, forty centuries look down upon you." Gentlemen, from the heights of past ages, countless worthies of our God-like profession point and beckon to a goal more elevated than that which attracts legislators and conquerors, Solons and Cæsars.

On motion of Dr. J. B. Biddle, the thanks of the Association were tendered to the President, Dr. Pope, for the able and eloquent address just delivered; and that a copy of it be requested for publication.

Dr. Hays stated that the Committee of Arrangements had agreed upon certain arrangements which would need the consent of the Association. He moved that the sessions of the Convention be held from 9 A.M. to 3 P.M., with one hour recess each day, from 12 M. to 1 P.M. Agreed to.

Dr. Hays then stated it was proposed by the Committee of Arrangements that the members of the Association should visit *this* afternoon the Pennsylvania Hospital for the Insane; on *Wednesday* afternoon, Girard College and Fairmount; on *Thursday* afternoon, Philadelphia Hospital, Blockley; on *Friday* afternoon, the Asylum for the Blind; and that arrangements had been made for conveying the members to those institutions.

Dr. Hays further stated that the Mayor of the City invited the Association to a reception at Independence Hall, at noon on Wednesday; and that invitations had been extended to the members to visit, at their convenience, the principal Public Institutions of the city, to all of which admission would be had on exhibiting their cards of membership.

Dr. J. B. Biddle moved that a recess be taken to allow the nomination of one from each State to serve as a committee to report nominations for permanent officers of the Association. Agreed to. The Association then took a recess, while the delegations assembled in various parts of the room.

Upon the re-assembling of the Association, Dr. D. F. Condie moved that all permanent members who, through want of notification, had not paid the amount for which they were assessed, but who had subsequently paid, be reinstated as permanent members of the Association.

Dr. Watson moved to amend the motion as follows:—

Resolved, That no member of the Association shall be deprived of his privilege as a permanent member by not contributing to pay the expenses of an annual meeting at which he is not present.

Dr. White, of Buffalo, moved that the whole subject be referred to a committee of three. Agreed to.

The Chairman appointed Dr. White, of Buffalo, Dr. Watson, of New York, and Dr. Condie, of Philadelphia.

The names of the different States were then called, each one reporting the name of its representative on the nominating committee as follows:

*Committee on Nominations.*

*Maine.*—A. J. Fuller.
*New Hampsaire*—Silas Cummings.
*Vermont.*—G. T. Elliott.
*Massachusetts.*—C. P. Fiske.
*Rhode Island.*—Jos. Mauran.
*Connecticut.*—P. A. Jewett.

*New York.*—John McCall.
*Pennsylvania.*—J. B. Biddle.
*New Jersey.*—Lewis Condict.
*Delaware.*—James W. Thompson.
*Maryland.*—Charles McGill.
*District of Columbia.*—Thomas Miller.
*Virginia.*—R. B. Welford.
*South Carolina.*—P. C. Gaillard.
*Georgia*—Richard D. Arnold.
*Alabama.*—P. H. Cabell.
*Tennessee.*—J. Berrien Lindsley.
*Kentucky.*—C. J. Blackburne.
*Ohio.*—R. Hills.
*Indiana.*—Joel Pennington.
*Illinois.*—J. V. Z. Blaney.
*Michigan.*—A. B. Palmer.
*Missouri.*—L. P. Perry.
*Iowa.*—J. E. Sandbourne.
*Wisconsin.*—J. B. Dousman.
*North Carolina.*—O. F. Manson.

Dr. F. C. Stewart, of New York, offered the following resolution :

*Resolved*, That the Nominating Committee be instructed to present three names to the Association as candidates for the office of President, who shall be elected by ballot; and the candidate who shall have the smallest vote, shall be withdrawn after the first ballot.

It was moved that the resolution be laid upon the table. Agreed to.

On motion of Dr. Atlee, it was Resolved, that the Nominating Committee be instructed to recommend a place for holding the next annual meeting of the Association.

Invitations to this effect were extended respectively by Dr. Pitcher, of Michigan, on behalf of the medical profession of Detroit; by Dr. Foster, of Tennessee, on behalf of the profession of Nashville; and Dr. Brainard, of Illinois, on behalf of the profession of Chicago. These invitations were, on motion, referred to the Committee on Nominations.

Dr. F. A. Ramsay moved that so much of the President's address as relates to the place of holding meetings of the Association, be referred to the Committee on Nomination.

Dr. D. D. Thompson, of Kentucky, moved that the regular order of business be dispensed with, to take up the amendments to the Constitution offered at the last annual meeting.

Several members objected, and the motion was lost.

The next business in order was the reception of "members by invitation."

On motion of Dr. Condie, the names of all "members by invitation" were referred to the Committee of Arrangement.

The President announced that the next business in order was the Reports of the different Standing Committees, appointed at the last annual meeting.

A report was received, through Dr. La Roche, from the Committee on Prizes. The report stated that the Committee had received six essays in competition for the Prize offered by the Association. But, although these essays evinced much ability and extensive learning, but one was decided to possess those qualities which deserved the award of the prize. The essay was entitled "Statistics of Placenta Prævia." The name of the author was announced as Dr. James D. Trask, of White Plains, New York. [Applause.]

On motion of Dr. Condie the report was accepted, and the Prize Essay was referred to the Committee on Publication.

Dr. Thomas Reyburn, Chairman of the Committee on Epidemics of Missouri, Iowa, Illinois and Wisconsin, submitted a voluminous report, the abstract of which occupied a long time in reading.

On motion, the report was referred to the Committee on Publication.

Dr. White, from the committee to whom was referred the resolutions in regard to the permanent members, submitted a report, recommending the adoption of the following resolutions:

*Resolved,* That upon no permanent member, who is not present at a meeting of the Association, shall be assessed the annual contribution; but no one shall be entitled to receive a copy of the printed *Transactions* unless he pay into the treasury a sum not less than the annual assessment paid by the delegates and permanent members in attendance; and that all the names of permanent members that have been left off the published list, be reinserted therein in the next volume of *Transactions.*

*Resolved,* That no assessment whatever shall be made against members by invitation, but that they also be entitled to a copy of the printed *Transactions* by paying the sum assessed upon delegates in attendance.

Dr. Sanford B. Hunt, of Buffalo, N. Y., from the Committee on the Hygrometrical State of the Atmosphere in various localities, and its influence on health, submitted a report, pending the reading of which the hour of adjournment arrived, and the Association adjourned to meet tomorrow morning at nine o'clock.

Second Day, May 2d.

The Association assembled at 9 o'clock. The minutes of the meeting of the preceding day were read and approved.

Dr. Condie moved that the Secretaries of this Association be requested to afford every facility possible to the reporters of the public press, to enable them to furnish full and accurate reports of the transactions.

Dr. Atlee, of Lancaster, asked and received permission to make a statement. At the meeting of the Association at Richmond, a resolution was passed, authorizing the appointment of a committee to procure a suitable stone for the Association to contribute to the Washington Monument, at Washington City, D. C. The Committee had made an assessment of $1 upon each member to purchase the stone and pay for the sculpture. Dr. Pierson, of Salem, Mass., had recommended as a design for the sculpture, "Hippocrates refusing the bribe offered by the King of Persia to visit the Court of that Prince, and give medical aid to the subjects of his Empire—when the great father of medicine said—'Tell your master that I am rich enough; that honor will not allow me to succor the enemies of Greece.'"

The sculpture was executed by a young man named John Augustus Beck, only twenty-two years of age. Eminent artists speak very highly of the work, which gives great promise of future eminence. Mr. Beck has been encouraged to go to Italy for study.

A resolution was offered, to the effect that no member should speak unless his name and residence were announced. Adopted.

Dr. J. B. Biddle, the Chairman of the Committee on Nominations, reported the following unanimous nominations :—

*President*—Geo. B. Wood, M.D., of Pennsylvania.

*Vice Presidents.*—Wm. M. Boling, of Alabama; Daniel Tilden, of Ohio; D. Humphreys Storer, of Massachusetts; Grafton Tyler, of the District of Columbia.

*Secretaries.*—Francis West, of Pennsylvania; R. C. Foster, of Tennessee.

*Treasurer.*—Caspar Wister, of Pennsylvania.

*Committee on Publications.*—Francis G. Smith, of Pennsylvania, Chairman; Francis West, of Pennsylvania; Samuel L. Hollingsworth, of Pennsylvania; H. S. Askew, of Delaware; Samuel Lewis, of Pennsylvania.

The committee also recommended, but not unanimously, Nashville, Tennessee, as the next place of holding the annual meeting.

On motion of Dr. Post, of New York, it was

*Resolved,* That the question of the confirmation of the officers, and of the place of meeting, be taken separately.

So much of the report as related to the nomination of officers, was then, on motion of Dr. Rogers, of Pennsylvania, adopted.

On motion, of Dr. White, of New York, it was

*Resolved,* That a committee of five be appointed to inform the President and Vice-Presidents elect, of the choice of the Association, and to conduct them to their respective seats.

The chair appointed Drs. J. M. Smith, of New York; Blackburne, of Kentucky; Homans, of Massachusetts; Rouse, of Illinois; and Frost, of South Carolina. The officers were then conducted to their seats by the committee.

On taking the Chair, the President, Dr. Wood, expressed in a neat speech, his thanks to the Association for the honor conferred upon him.

On motion of Dr. Biddle, of Pennsylvania, the thanks of the Association were tendered to the retiring President, Dr. Charles A. Pope, for the able and efficient manner in which he had discharged his duties.

Dr. Post, of New York, moved that the next place of meeting be the City of Washington.

Dr. Watson, of New York, moved, as an amendment, to substitute the City of Detroit for Washington.

Dr. L. A. Smith, of New Jersey, moved, as a further amendment, the substitution of Nashville for Detroit.

After some discussion, Detroit was selected as the place for the next meeting.

Dr. Foster, one of the newly elected Secretaries, tendered his resignation, which was, on motion, accepted.

Dr. Brodie, of Michigan, was then chosen to fill the vacancy.

The further consideration of the report of Dr. Sanford B. Hunt, of New York, upon the Hygrometrical State of the Atmosphere in various localities, was resumed.

Dr. Hunt read an abstract of his report, which was very interesting, and was listened to with a great deal of attention. It abounded in facts and statistics, illustrating the influences of the changes in the atmosphere, particularly with reference to epidemics, and deduced several laws as governing a certain class of epidemics.

On motion, the report was referred to the Committee on Publication.

Dr. Frank H. Hamilton, of Buffalo, N. Y.; then submitted a report upon the Frequency of Deformities in Fractures. The report was accompanied with voluminous statistics. The main object of the report was the demonstration of the impossibility of the union of fractures without distortion.

Dr. Hamilton said he had a word to say, but not in connection with the report. It was necessary to do something to arrest the frequency of the prosecutions for mal-practice. The frequency of these prosecutions no longer surprised the members of the profession. They had become familiar. What occasions this frequency? Is it because there are jealous and designing men among the profession? There are a few, no doubt. But, upon the whole, no profession stands by itself as well as the medical profession. Is it the fault of the lawyers? Lawyers occasionally might have something to do with encouraging the prosecutions; but this would not account satisfactorily for the evil complained of. The speaker thought the reason could be found in the imperfection of the art, and the reluctance of the profession to admit those imperfections. Members would assure the world, for instance, that a fractured femur could be united without shortening the limb—which was simply impossible. He trusted that the profession would be wiser in the future, and acknowledge that they could not perform impossibilities. Even that city of medical science, Philadelphia, had not produced a book which could instruct physicians how to unite a fractured femur without shortening the limb.

The report was referred to the Committee on Publications, and the Committee, at the request of Dr. Hamilton, continued.

Dr. Charles Hooker, of New Haven, Conn., submitted a report upon "Diet for the Sick." The report lays down laws for the government of diet under various diseases, and specifies particular articles which may be given with benefit. Referred to the Committee on Publications.

On motion, a resolution was adopted, returning the thanks of the Association to the retiring Vice Presidents, Secretaries and Treasurer.

An invitation to visit the Central High School to-day, at noon, was accepted.

A resolution was adopted, returning thanks to railroad companies that had given commutation tickets to members of the Medical Association.

On motion of Dr. Hintze, it was

*Resolved,* That a committee of one from each State be appointed by the chair to use their influence with the railroad and steamboat companies, in their respective States, to issue commutation tickets to the delegates to the American Medical Association, and their immediate families, who may design attending the meeting of the Association in May, 1856.

The following were appointed the committee:—

*Maine.*—E. R. Peaslee.
*New Hampshire.*—Josiah Crosby.
*Vermont.*—J. Perkins.
*Massachusetts.*—J. Homans.
*Rhode Island.*—Joseph Mauran.
*Connecticut.*—P. A. Jewett, *Chairman.*
*New York.*—James P. White.
*Pennsylvania.*—Francis West.
*New Jersey.*—Lyndon A. Smith.
*Delaware.*—James W. Thompson.
*Maryland.*—F. E. B. Hintze, *Sec.*
*District of Columbia.*—Grafton Tyler.
*Virginia.*—B. R. Wellford.
*North Carolina.*—O. F. Manson.
*South Carolina.*—R. F. Michel.
*Georgia.*—Richard D. Arnold.
*Alabama.*—William M. Boling.
*Tennessee.*—J. B. Lindsley.
*Kentucky.*—C. J. Blackburne.
*Ohio.*—Daniel Tilden.
*Illinois.*—Rudolphus Rouse.
*Missouri.*—Charles A. Pope.
*Iowa.*—J. E. Sanborn.
*Wisconsin.*—J. R. Bartlett.
*Michigan.*—Zina Pitcher.

On motion, the Association adjourned to Independence Hall.

*The Medical Association at Independence Hall.*—About five minutes past twelve o'clock, the members of the Association entered Independence Hall. A number of ladies were already present, and the Hall was soon crowded to its utmost extent. [We have not space to publish the eloquent speeches of Dr. Hays and Mayor Conrad.]

AFTERNOON SESSION, 1 P. M.

On motion of Dr. Davis, of Illinois, the thanks of the Association were tendered to the Mayor of Philadelphia for the very cordial manner in which he had received the American Medical Association at Independence Hall, and that a copy of his eloquent speech be published in connection with the proceedings of the Association.

On motion of Dr. Askew, of Delaware, the rules of order were suspended for the purpose of allowing Dr. J. W. Thompson, of Delaware, to offer the following preamble and resolutions:—

*Whereas,* That as few subjects of greater interest and importance could be presented to the consideration of the American Medical Association, now representing most of the States and Territories of the Union, than the attainment of a correct Medical Topography of each, with a history of its prevailing fevers and most successful treatment of the same, therefore be it

*Resolved,* That with this view and conviction, this Association appoint a Special Committee for each State and Territory represented, of ——, members whose duty it shall be to report upon its medical topography epidemic fevers and most successful treatment thereof, and that the same shall continue to hold their office for three years.

*Resolved,* That as other States and Territories not now represesented, become so by delegates duly appointed to this National Association; that similar committees shall be appointed for like purpose, and that they also shall hold their office for three years.

*Resolved,* That in the appointment of gentlemen of education and experience in the affairs of their own State, we have the best guarantee that the important objects we seek will be more satisfactorily accomplished, and the profession as well as the public interest will thereby be better served.

*Resolved,* That the committees heretofore appointed by this Association, at its session in Charleston, for a similar object, be, and the same are hereby discharged.

On motion of Dr. Askew, of Delaware, the foregoing preamble and resolutions were ordered to lie on the table and made the special order for to-morrow at 10 o'clock.

The Committee on Publication submitted their report. The seventh volume of the proceedings of the Association was issued last November, 1000 copies being published, at an expense of $1806 42; 781 copies have been sold or furnished to members of the Association; 35 were given to editors of medical journals, and 184 remain on hand.

In view of the delay in publication which is liable to be occasioned by the reference to the Committee on Publication, of reports and other papers of which abstracts alone are presented to the Association, the committee recommended for adoption the following resolution:

*Resolved,* That hereafter, beginning with the session for 1856, no report or other paper shall be entitled to publication in the volume for the year in which it shall be presented to the Association, unless it be placed in the hands of the Committee on Publication on or before the first of June.

Dr. Biddle, of Pennsylvania, offered the following resolution, which was adopted:—

*Resolved,* That the thanks of the Association are eminently due to the Committee of Publication for the faithful and highly satisfactory manner in which their arduous and responsible duties have been discharged.

The Treasurer submitted his annual report:

| | | |
|---|---|---|
| Balance in the Treasury at last account, . . | $293 | 99 |
| Assessment and Sales of Transactions, . . | 2722 | 31½ |
| Prize Fund, . . . . . . . . . | 200 | 00 |
| Total, . . . . . . . . . . | 3216 | 30½ |
| Balance in Treasurer's hands at present, . . | $1115 | 26 |

A resolution was adopted, appropriating the sum of $1000 to pay for the stone for the Washington Monument.

Dr. Duhamel, of Washington City, offered a preamble and resolutions returning the thanks of the Association to those Senators and Representatives who took an interest in procuring the passage of the Quarantine bill, the object of which is the better prevention of the introduction of diseases into the country.

The resolutions were adopted.

The Secretary read a paper from Dr. Wm. H. Byford, of Evansville, Indiana, upon Scrofula. This paper gives an account of the nature of the disease, its varieties, causes, effects, and treatment. Referred to the Committee on Publications.

Dr. N. S. Davis, of Chicago, Ill., read a paper upon "The Nutritive Qualities of Milk, and the Influence produced thereon by Pregnancy and Menstruation, in the Human Female, and by Pregnancy in the Cow; and also on the question whether there is not some mode by which the nutritive constituents of milk can be preserved in their purity and sweetness, and furnished to the inhabitants of cities in such quantities as to supersede the present defective and often unwholesome modes of supply." In this report, the various methods of preserving milk are all investigated and explained, and the preference given to that discovered by a gentleman of Rochester, New York. By this method, milk had been preserved for twelve months, with all its nutritive qualities. The solidified milk, prepared by that gentleman, was decided to be the article that had long been a desideratum. Referred to the Committee on Publications. The committee was continued.

The hour of adjournment having arrived, on motion, the Association adjourned.

THURSDAY, MAY 3, 1855.

The Association assembled at 9 A. M., the President, Dr. Wood, of Pennsylvania, in the chair.

The minutes of the last meeting were read and approved.

The President stated that it had been usual to refer the appointment of the various standing committees to the Committee on Nominations. Whereupon, on motion of Dr. Watson, of New York, it was

*Resolved*, That these appointments be referred to that Committee.

Dr. Hays, of Pennsylvania, presented an invitation from Dr. Ducachet, Rector of St. Stephen's Church, to visit that church and view the "Burd Monuments" therein.

The invitation was accepted, and 12 M. to-day appointed as the time for the visit, and the thanks of the Association directed to be tendered to Dr. Ducachet.

Dr. Hays also presented an invitation from the Principal and Managers of the Training School for Idiotic and Feeble-minded Children, to visit that Institution on Friday afternoon next. Invitation accepted, and the thanks of the Association directed to be returned for the invitation.

A letter was read from Dr. E. B. Haskins, of Clarkesville, Tennessee, the Chairman of the Committee on Microscopical Investigations of Malignant Tumors, stating that he had not the means of carrying out the object of the Committee, and asking to be discharged, which was agreed to.

A communication was read from Dr. Reyburn, of Mississippi, suggesting that the District, composed of Missouri, Illinois, Wisconsin, and Iowa be divided, and also offering his resignation as Chairman of the Committee appointed to report upon the epidemic of those States, and on motion of Dr. Askew, of Delaware, it was

*Resolved*, That so much of said communication as refers to the division of districts be referred to the Committee on Nominations, and that the resignation of Dr. Reyburn be accepted.

The Committee on plans of Organization for State and county societies, Dr. A. B. Palmer, of Michigan, chairman, was called, when the chairman stated that the Committee was not prepared to report.

Committee on Medical Literature, Dr. R. J. Breckenridge, of Kentucky, Chairman, was called, when a letter was read from the chairman, stating his excuses for not having his report prepared, and, after considerable discussion, the committee was continued.

Committee on Medical Education, Dr. Wm. H. Anderson, of Alabama, Chairman, was called on, when a letter was read from the chairman, stating his reasons for not having his report ready, and asking to be continued.

After some discussion upon the general question of the propriety of continuing committees who failed to present their reports in time, on motion of Dr. J. L. Atlee, of Pennsylvania, it was

*Resolved*, That the further consideration of the subject be postponed in order to receive the report of the Committee on Nominations.

Dr. J. B. Biddle, Chairman of this Committee, made the following report:—

The Committee on Nominations, to which was referred the resolutions in reference to the appointment of a Committee on Medical Topography and Epidemics, report the resolutions, with a recommendation that they be adopted.

They recommend the following gentlemen to compose the committee of one from each State and Territory, to be appointed in accordance with the resolutions, viz:—

*Maine*—J. C. Weston, of Bangor.
*New Hampshire*—Edmund R. Peaslee, Dartmouth College.
*Vermont*—Joseph Perkins, of Castleton.
*Rhode Island*—Joseph Mauran, Providence.
*Connecticut*—Charles Hooker, New Haven.
*Massachusetts*—George C. Shattuck, Boston.

*New York*—Joseph M. Smith, New York.
*New Jersey*—Lyndon A. Smith, Newark.
*Pennsylvania*—Jacob A. Gemmil, Huntingdon County.
*Delaware*—James W. Thompson, Wilmington.
*Maryland*—Peregrine Wroth, Chestertown.
*Georgia*—John F. Posey, Savannah.
*Virginia*—P. F. Peebles, Petersburg.
*District of Columbia*—Thomas Miller, Washington.
*South Carolina*—D. J. Cain, Charleston.
*North Carolina*—O. F. Manson, ———
*Kentucky*—Wm. L. Sutton, Georgetown.
*Tennessee*—E. B. Haskins, Clarkeville.
*Louisiana*—E. D. Fenner, New Orleans.
*Minnesota*—J. H. Murphy, St. Anthony's Falls.
*Ohio*—G. Mendenhall, Cincinnati.
*Mississippi*—T. J. Grafton, Rodney.
*Missouri*—S. B. Alleyne.
*Michigan*—J. H. Beech, Cold Water.
*Alabama*—S. W. Chanton, Warsaw.
*Illinois*—John Evans, Chicago.
*Indiana*—Vierling Kersey, Milton, Wayne County.
*Wisconsin*—Alfred L. Castleton, Delafield.
*Iowa*—E. A. Arnold, Davenport.
*U. S. Navy*—Thomas Dillard, Philadelphia.
*U. S. Army*—Clement A. Finley.

The committee report that, in their opinion, it is inexpedient to make any change in the Constitution in regard to the time and place of meeting of the Association.

A number of Special Committees were appointed, and the following

STANDING COMMITTEES.

*Committee on Arrangements*—Dr. Zina Pitcher, of Detroit; Dr. Moses Gunn, do.; Dr. A. S. Leland, Dr. Moses Stewart, Dr. Peter Klein, Dr. J. A. Brown.

*Committee on Prize Essays*—Dr. A. B. Palmer, of Michigan; Dr. Samuel Denton, Dr. A. R. Terry, Dr. Adam Sager, Dr. S. H. Douglass, Dr. Corydon La Ford, Dr. E. Andrews.

*Committee on Medical Literature*—Dr. P. C. Gaillard, of South Carolina; Dr. N. P. Monroe, of Maine; Dr. James Couper, of Delaware; Dr. R. Hills, of Ohio; Dr. A. Coffin, of South Carolina.

*Committee on Medical Education*—Dr. J. Berrien Lindsley, of Tennessee; Dr. J. B. Flint, of Kentucky; Dr. P. H. Cabell, of Alabama; Dr. George Hayward, of Massachusetts; Dr. E. B. Smith, of Missouri.

On motion, the report of the committee was adopted.

The subject of the report of the Committee on Medical Education was taken up, and after considerable discussion Dr. Lindsley, the chairman of the newly-appointed committee on the subject, asked leave to resign, and that Dr. Anderson, of Alabama, chairman of the former committee, be appointed in his place.

Dr. Lindsley's resignation was accepted, and Dr. Anderson appointed in his place.

A letter from Dr. W. Hooker, Chairman of the Committee on the Epidemics of New England and New York, was read, in which he points out the defects of the present organization of the Committee on Epidemics, and asking to be discharged from further consideration of the subject.

On motion of Dr. Hays, the letter was referred to the Committee on Nominations.

Dr. F. H. Hamilton, of New York, asked and obtained leave to make some remarks on fracture of the clavicle, supplementary to his report made yesterday.

The special order of the day, the resolutions in reference to the appointment of Committees on Medical Topography, offered by Dr. Thompson, of Delaware, was taken up.

After some remarks by Dr. Thompson, advocating their adoption, Dr. Askew, of Delaware, offered the following additional resolution, which was accepted by Dr. Thompson :—

*Resolved,* That all reports on the Medical Topography and prevailing diseases of the States, shall, to entitle them to be received by this Association and published in the Proceedings, be first approved by the medical societies of the State or Territory where such Societies exist, and to which State or Territory such report refers.

The preamble and resolutions were then adopted.

Dr. J. G. Orton, of Binghamton, New York, read a series of resolutions which he had prepared in reference to the same subject. They were as follows :—

*Resolved,* That each County Medical Society (or, in parts of the country where such societies have not been established, any duly organised medical association), be invited to amend their constitutions by attaching thereto the following article : "It shall be the duty of each member of this Society to keep a faithful record of the diseases which may fall under his observation during each month, according to the classification adopted by this Convention in May, 1847, stating the age, sex, occupation and nativity of the patients, the average duration of the disease, and, finally their recovery or death, and to report the same in writing to the Secretary, on or before the first day of February of each year, who shall transmit a digest thereof to the State Medical Society, and also to the appropriate committee appointed by the American Medical Association for its reception."

*Resolved,*—That each incorporated Hospital, Infirmary, and Asylum, be invited to furnish a copy of their annual reports for the use of the committees of their respective States.

*Resolved,* That the State Committee appointed by this Association to report upon the prevailing diseases of their respective localities, shall receive and arrange a digest of the reports transmitted by them to the Secretaries of the various county societies, and report the same at the annual meeting of this Association.

*Resolved*, That the first day of January be the time fixed at which the object of these resolutions shall be carried into effect, and that the several county societies and associations be requested to amend their constitutions as heretofore recommended, at as early a date as practicable, and to report to the State Committee their willingness or unwillingness to acquiesce in the request of this Association.

On motion, the resolutions in reference to this subject were referred to the Committee on Nominations.

On motion of Dr. J. L. Atlee, of Pennsylvania, the Chief Justice of the Stace, the Hon. Ellis Lewis, who was present, was invited to take a seat on the platform.

The President stated that Dr. D. J. Cain, appointed to report on the epidemics of South Carolina, Florida, Georgia and Alabama, was confined at home by sickness.

The Committee on the Epidemics of Tennessee and Kentucky, not being ready to report, was continued.

A letter was read from Dr. Fenner, appointed to report on the epidemics of Louisiana, Mississippi, Arkansas, and Texas, stating that his report was in progress.

On motion, the committee was continued.

Dr. D. Francis Condie, to whom was referred the subject of tubercular disease, stated that he had been engaged for a period of nearly three years in examining and arranging a report on this subject. The report will occupy at least five hundred pages, and one reason which has caused it to swell to so great an extent, is, that he found it necessary to contradict a large number of statements and reports on the subject. He hoped that the Association would excuse him in not being quite ready with his report. He had prepared a digest of its contents, which in fact is merely an index to the whole, and would not of itself repay for the time used in reading it. He expressed a desire that the Association would excuse him.

Dr. Altee moved that Dr. Condie do just as he please, with his paper, without any obligation he may consider himself under to the Association. The motion was agreed to.

The Committee on Dysentery, Dr. Henry Taylor, of Mt. Clemens, Michigan, chairman, presented, through Dr. Beech, an abstract of the report which was read, and, on motion, the report was referred to the Committee of Publication.

The Committee on Hydrophobia and the Connection of the Season of the Year with its Prevalence, Dr. T. W. Blatchford, of New York, chairman, presented a partial report, which was read, and, on motion of Dr. Smith of New York, the committee was continued.

The hour for recess having arrived, Dr. Hays, on behalf of the Committee of Arrangement, begged to state that St. Stephen's Church, in which were the beautiful monuments by Steinhauser to the Burd family, was open for the inspection of the members of the Association, as were also the Galleries of the Academy of Fine Arts, the Museum of the Academy of Natural Sciences, and the Museums of the different Medical Colleges, and invited the members to visit them. He also reminded the members that this was the time appointed to visit the High School, and such as desired to do so could visit that useful Institution.

May 3—1 P. M.

Association reassembled, the President, Dr. Wood, in the Chair.

Dr. R. D. Mussey, of Ohio, the Committee to examine into, and report upon, the effects of Alcoholic Liquors upon the system in health and disease, read his report, which was, on motion, referred to the Committee on Publication.

On motion of Dr. Thomas, of Maryland, the resolution referring the report on Medical Literature to the Committee of Publication was reconsidered, when, on motion of Dr. Gibson, of Virginia, the further oonsideration of this subject was postponed until to-morrow.

On motion of Dr. Jewett, of Connecticut, it was

*Resolved*, That a Committee of one from each State be appointed to report upon a uniform system of marriages, births and deaths, which resolution was referred to the Committee of Nominations, to report the names of the Committee.

The hour of adjournment having arrived, Dr. Hays, on behalf of the Committee of Arrangements, stated that omnibuses would be in waiting at 4½ P. M., to convey the members and the ladies of their party to the Philadelphia Hospital, Blockley.

The Association then adjourned.

Friday, May 4, 1855.

The Association met this day at 9 A. M., the President, Dr. Wood, of Philadelphia, in the Chair.

The minutes of the last meeting were read and approved.

Dr. Atlee offered a resolution that Dr. Breckenridge's report on Medical Literature be referred to a special committee, to be read, and if approved by said committee, then that it be referred to the Committee on Publication. The motion was lost.

A motion was then made and adopted, that the Committee on Medical Literature be continued to report at the next meeting of the Association.

Dr. Hayward, of Boston, offered the following, which was adopted unanimously:—

*Resolved*, That the thanks of this Association be presented to his Honor the Mayor, and the other officers of the city government, and the citizens and physicians of the city of Philadelphia, for their kind and continued attentions, and munificent hospitality to the members of this Association during its present session.

Dr. J. L. Atlee, of Pennsylvania, presented and read a report, on behalf of the committee appointed at the last annual meeting of the Association, and to which was referred the essay of Dr. Phelps, of New York, on "Religion as an Element in Medicine, or the Duties and Obligations of the Profession," and also the communication of Dr. R. S. Bailey of South Carolina, on the same subject, with instructions to consider and report on the expediency of publishing the same. The report was accompanied by the following resolution:—

*Resolved*, That it is inexpedient to publish in our *Transactions* the Essay of Dr. Phelps.

The committee state that, having expressed their sentiments on the subject, they beg to be discharged from the further consideration of Dr. Bailey's paper.

After a brief discussion, the previous question was demanded and sustained, and the report was accepted, the accompanying resolution adopted, and the request to be discharged granted.

A printed paper on Cholera, by Dr. Knapp, of Cincinnati, was presented, but as it had been already published, it was not deemed expedient to occupy the time of the Association by having it read.

Dr. J. L. Atlee, of Pennsylvania, stated that the Academy of Fine Arts had offered to take charge of the stone for the Washington Monument, and offered a resolution that a vote of thanks be tendered to the Managers of the Academy of Fine Arts, for their liberal offer to take charge of the stone for the Washington monument, until its delivery at Washington, which was adopted.

Dr. Charles Hooker, of Connecticut, offered the following amendment to the Constitution, which, under the rules, lay over until the next annual meeting of the Association.

"Any permanent member who shall not pay for the published *Transactions* for three successive years, shall be considered as withdrawn."

Dr. N. S. Davis, of Chicago, Illinois, submitted the following preamble and resolutions :—

*Whereas*, The present mode of conducting the annual meetings of the Association affords but little opportunity for the discussion of truly scientific questions and papers, and

*Whereas*, This has been regarded as a serious defect in the operation of our organization, impairing its scientific character, therefore

*Resolved*, That the daily sessions of the Association, during each annual meeting, be divided into two parts—the first to terminate at an hour not later than 12½ o'clock P. M., each day, and to be devoted as heretofore, to the general business of the Association—the second, consisting of all the time which it is deemed advisable to remain in session each day, after 12½ o'clock, P. M., to take the character of a scientific session, and to be devoted exclusively to the discussion of questions relating to the science and art of medicine.

*Resolved*, That the Association, in its capacity of a scientific session, having no power to act on any subject except of a scientific character, may continue in session, whenever thought advisable, a longer period than in its more general capacity.

*Resolved*, That the foregoing preamble and resolutions be referred to the Committee of Arrangements, with instructions to report on the same at the commencement of the next annual session.

Dr. C. G. Comegys, of Ohio, offered the following preamble and resolutions, which were, on motion, referred to the Committee on Nominations.

*Whereas*, The American Medical Association, though devoting itself ardently to the interest of medical science, is unable to accomplish all its aims for the promotion of the usefulness of the profession in society without the aid of legislation, therefore

*Resolved,* That the members of the medical profession throughout the Union be urgently requested to take immediate and concerted action for petitioning their several legislative bodies to establish offices for the collection of vital statistics.

*Resolved,* That for the purpose of securing an ample number of petitioners for this object, this Association suggests that every member of the profession shall endeavor to secure the names of his personal friends.

*Resolved,* That a copy of the above resolutions be transmitted at once to the several State societies.

Dr. A. J. Semmes, of Washington, D. C., offered the following, which was adopted :—

*Resolved,* That a committee of three be appointed to report to the Association, at its next annual meeting, what measures should be adopted to remedy the evils existing in the present methods of holding coronor's inquests, by incompetent persons, by which the lives and liberties of the innocent may be jeopardized, and the ends of justice frusstrated.

The President appointed Dr. A. J. Semmes, of Washington; Grafton Tyler, D. C.; and Dr. D. F. Condie, of Pennsylvania, that committee.

Dr. F. C. Stewart, of New York city, offered the following resolutions, which were unanimously adopted :—

*Resolved,* That the thanks of the American Medical Association be, and they are hereby tendered to the Committee of Arrangements for the liberal and cordial reception extended to its members during the present session.

*Resolved,* That a similar acknowledgment is eminently due, and also unanimously extended, to the officers, trustees, and managers of the several public and private institutions of the city and vicinity, which have been thrown open to the inspection of the members of the Association, the visitation of which has been a source of mingled satisfaction, and the management of which manifests the faithful and zealous care of those to whose guardianship they have been entrusted.

Dr. Biddle, Chairman of the Committee on Nominations, to whom was referred the resolution of Dr. Jewett, "that a committee of one from each State be appointed by this Association to report upon a uniform system of Registration of Marriages, Births, and Deaths," made a report, recommending the adoption of the resolution, and present the following list of names to constitute said committee : —

Drs. R. W. Wilson, of Hartford, Conn., Chairman ; G. S. Palmer, of Gardiner, Me.; Silas Cumming, Fitz-William, N. H.; G. T. Elliot, Woodstock, Vt.; Edward Jarvis, Dorchester, Mass.; Joseph Mauran, Providence, R. I.; Jno. H. Griscom, New York, N. Y.; Henry Carpenter, Lancaster, Pa.; O. H. Taylor, Camden, N. J.; Lewis P. Bush, Wilmington Del.; A.Snowden Piggott, Baltimore, Md.; David H.Tucker, Richmond, Va.; —— Pitman, Tarboro, N.C.; Harry Lindsley, Washington, D. C.; Jno. D. Dawson, Charleston, S. C.; R. D. Arnold, Savannah, Ga.; A. Lopez, Mobile, Alabama; J. Jones, New Orleans, La.; R. C. Foster, Nashville, Tenn.; C. J. Blackburne, Covington, Ky.; Jno. Dawson, Columbus, Ohio; Edmund Murphy, New Harmony, Indiana; A. D. Stebbins, Detroit, Mich.; J. B. Z. Blaney, Chicago, Ill.; Geo. D. Wilbur, Mineral Point, Wis.; Wm. M. McPheeters, St. Louis, Mo.; J. D. Elbert, Keosaqua, Iowa; Jno. H. Murphy, Falls of St. Anthony, Minnesota.

Mississippi and Arkansas were left blank.

Dr. J. L. Atlee, of Pennsylvania, offered the following resolutions, which were adopted:

*Resolved*, That to secure efficient teaching in medical schools, where the prime object is to enforce practical precepts, a large degree of union and harmony must exist among the teachers, and confidence be reposed in them by their pupils.

*Resolved*, That any such unnatural union as the mingling of an exclusive system, such as homœopathy, with scientific medicine, in a school, setting aside all questions of its untruthfulness, cannot fail, by the destruction of union and confidence and the production of confusion and disorder, unsettling and distracting the mind of the learners, to so far impair the usefulness of teaching as to render every school adopting such a policy unworthy the support of the profession.

Dr. Foster, of Tennessee, offered the following resolution, which, on motion, was laid on the table:

*Resolved*, That the State Medical Societies be requested to hold their annual meetings just one month before the meeting of the American Medical Association.

Dr. Clendennin, of Cincinnati, Ohio, offered the following:—

*Resolved*, That no State or local society shall hereafter be entitled to representation in this Association that has not adopted its code of ethics.

*Resolved*, That no State or local society that has intentionally violated or disregarded any article or clause in the code of ethics, shall longer be entitled to representation in this body.

This brought up before the Association the case of the Ohio State Medical Society, which, at its late annual meeting, on motion of Dr. Grant, "*Resolved*, That it is not derogatory to medical dignity, or inconsistent with medical honor, for medical gentlemen to take out a patent right for surgical or medical instruments." That resolution is in direct opposition to § 4 of Art. I. Chap. II. of the Code of Ethics, which says: "Equally derogatory to professional character is it, for a physician to hold a patent for any surgical instrument," &c.

A member from Ohio, Dr. Hills, defended the State Medical Society, deprecating any action upon the subject. He said that the obnoxious resolution had been carried by a very small majority; that the Society, as a body, accepted in full the Code of Ethics of the Medical Association, and could *only* have meant by its resolution an *erroneous interpretation* of the code, and not an opposition to its requirements.

Dr. Lemoine, of St. Louis, stated that a resolution on this subject had been made at St. Louis.

Dr. Lajus detailed, that at the St. Louis meeting, "Dr. J. E. Nagle, of Kenton, Ohio, proposed that the words, 'for any surgical Instrument,' in Chap. II., Art. I., § 4, of the Code of Ethics be erased. The proposition was not adopted." (Vide Transactions.) Dr. Lajus remarked, that the sense of the Association on this subject had been thus fairly and fully obtained, and that too by a member from *Ohio;* that the State Medical Society, by its resolution, had therefore, not only violated the code of ethics, but had also acted in opposition to the special action of the Association at its meeting in St. Louis, in confirmation of a section of that code.

Dr. Cock, of New York, made some eloquent remarks on the value of the code of ethics, and the importance of the Association upholding it as its safeguard, allowing no dereliction of its laws to pass unnoticed.

Several members advocated the removal *at once* from the erring Society of the right of representation.

Dr. Miltenberger, of Maryland, offered the following preamble and resolutions, to be appended to the resolutions of Dr. Clendennin:—

*Whereas,* It has been brought to the notice of the American Medical Association that the State Medical Society of Ohio violated, at their last meeting, one of the articles of its Code of Ethics. Therefore,

*Resolved,* That the Secretary of this Association be directed to inform the officers of that Society, that unless such action be rescinded, they cannot hereafter be represented in this Association.

Dr. J. L. Atlee, of Pennsylvania, offered an amendment to the foregoing preamble, the addition thereto of the particular obnoxious action alluded to, viz: the adoption of a resolution, "That it is not derogatory to medical dignity, or inconsistent with medical honor, for medical gentlemen to take out a patent right for surgical or medical instruments."

The amendment was accepted by Dr. Miltenberger, and the resolutions of Dr. Clendennin, with the preamble and resolutions of Dr. Miltenberger, and as amended by Dr. Atlee, were adopted. The resolutions as adopted are as follows:—

*Resolved,* That no State or local society shall be hereafter entitled to a representation in this Association, that has not adopted the code of ethics.

*Resolved,* That no State or local society that has violated or discarded any article or clause in the Code of Ethics, shall longer be entitled to a representation in this body.

*Whereas,* It has been brought to the notice of the American Medical Association that the State Medical Society of Ohio violated, at their last annual meeting, one of the articles of its Code of Ethics by adopting a resolution, "That it is not derogatory to medical dignity or inconsistent with medical honor for medical gentlemen to take out a patent right for surgical or medical instruments," therefore,

*Resolved,* That the Secretary of this Association be directed to inform the officers of that society, that, unless such action be rescinded, they cannot hereafter be represented in this Association.

Dr. A. Stillé, of Pennsylvania, offered the following resolutions, which were adopted:—

*Resolved,* That a special committee of five be appointed to report at the next meeting of the Association upon the following question :—

Might not the present system of repeating the same lectures to the same classes during two successive terms be usefully modified by extending the lectures of each chair over two sessions, so as to embrace a systematic and complete discussion of each of the following subjects :—

1. Special, Regional, and General Anatomy, including illustrative references to Morbid Anatomy.
2. Inorganic, Organic, and Pharmaceutical Chemistry and Toxicology.
3. General and Human Physiology, Hygiene, and Medical Jurisprudence.
4. Medical Botany, Materia Medica, Therapeutics.
5. General Pathology, Morbid Anatomy (systematic), Practice of Medicine.
6. General Surgical Pathology, or Institutes of Surgery, Mechanical Operative and Medicinal Surgery.
7. Obstetrics, Diseases of Women, Diseases of Children.
8. Hospital Clinical Medicine and Surgery.

*Resolved,* That the committee, at an early day, address the several medical colleges in regard to the proposed plan of instruction, requesting from them an official expression of opinion upon its merits and feasibility.

On motion of Dr. Pitcher, it was

*Resolved,* That the committee under the above resolution be appointed by the President, and that he be allowed to do this after the final adjournment of the Association.

The President appointed the following committee under the preceding resolutions :—

Dr. Alfred Stillé, of Philadelphia, chairman; Professor Samuel Jackson, of Philadelphia; Dr. John Bell, of Philadelphia; Dr. John Watson, of New York; and Dr. J. L. Cabell, of Charlottesville, Virginia.

Dr. Levi D. Scheets, of Ohio, offered the following alterations to the Constitution, which, under the rule, lay over until the next meeting :—

*Whereas,* It has been found necessary to devise some means of elevating the standard of education, both professional and general, among medical men, by those who are best acquainted with the sad and lamentable deficiency which prevails in this respect among a large mass of the profession, and particularly in the Western States. And,

*Whereas,* Efforts to this effect at home, have been opposed on the ground that more was exacted by such as took an active part in the matter, than is required by the "American Medical Association." And,

*Whereas,* It is believed that the national Society can exert a beneficial influence over the whole country. Therefore,

*Resolved,* That the Constitution of the Association be so amended as to require all delegates, before being allowed a seat in said Association, to satisfy the proper authorities that the societies which they represent, require *graduation* as the *sine quâ non* to membership therein, and that no person can become a permanent member, a member by invitation, or can be received as a delegate from any other body, unless he be a graduate of some respectable medical school.

*Resolved*, That the editors of the various medical journals of the United States be requested to publish the foregoing, so that an interest may be awakened on this subject, and that societies may be prepared to comply with the above requisitions in case they meet the approval of this Association.

Dr. Corson, of New York, asked and obtained leave to read a brief abstract of a paper "On the Influence of Lead on the Heart's Action, and the Importance of the Study of the Cardiac Impulse in Disease, generally founded on a Series of Observations."

On motion, it was referred to a special committee of three, with instructions, if they approve of it, to transmit it to the Committee of Publication for insertion in the Transactions.

Drs. Charles D. Davis, Isaac Wood, and F. Campbell Stewart were appointed the committee.

Dr. Thomas, of Baltimore, read a paper on a simple and easy method of applying nitrate of silver to the air-passages, contrived and employed by the author's brother, Dr. John Chew Thomas, about six years since. It consists in applying a stick of caustic lightly to a fine grindstone or emory wheel made to revolve with great velocity. The caustic is thus thrown off in an impalpable powder, which may be inhaled by the patient sitting before the stone.

On motion of Dr. Emerson, the paper was referred to a committee of three, with instructions, if they deem it worthy of insertion in the Transactions, to transmit it to the Committee of Publication.

Drs. Emerson, of Philadelphia, Miltenberger, of Maryland, and R. P. Thomas, of Philadelphia were appointed the committee.

Dr. Atlee offered the following resolution, which was adopted :—

*Resolved*, That the subject of the contagiousness or non-contagiousness of cholera be especially recommended to the attention of the State Committee on Epidemics, State Topography, &c.

The proposition offered at the last annual meeting by Dr. S. D. Gross, of Kentucky, to amend that part of the Constitution which relates to the election of officers, so that the election shall take place immediately before the adjournment of each meeting, instead of immediately after its commencement, was called up, and, on motion, indefinitely postponed.

The proposition offered at the last annual meeting by Dr. F. A. Ramsay, of Tennessee, that the constitution be so amended as to dispense with the Nominating Committee, and the duties of such committee, was, on motion, rejected.

Dr. N. B. Ives, of Connecticut, proposed the following amendment to the Constitution, to be appended to the end of the second article :—

"The Association shall, at all times, have power to punish any of its members by reprimands, suspension, or expulsion, upon a three-fourths vote of the members present at any meeting, at which not less than one hundred are in attendance."

Dr. Dorset, of Virginia, offered the following amendments to the Constitution :—

*Resolved,* That with a view of adding dignity and influence to our body, and conveying a more adequate idea of its national representative character, the title "National Medical Congress" be substituted for that of "American Medical Association," and that we hold our sessions at least once in three years in Washington, D. C.

Dr. A. M. Pollock offered the following resolution :—

*Resolved,* That no organization or institution, entitled to representation in this Association, shall be considered in good standing which has not adopted its code of ethics.

Dr. Lemoine, of Missouri, called up the amendment to the Constitution offered last year by Dr. Byford, to change the name of the "Committee on Epidemics" to "Committees on Prevailing Diseases."

On motion, the proposed amendment was rejected.

Dr. Hays stated that the Mayor of the city, at the suggestion of some citizens who were anxious that the account of the reception at Independence Hall should be preserved in a more durable form and better fitted for distribution than in a newspaper report, had determined to publish it in a neat pamphlet, which would be ready for distribution to members of the Association to-morrow morning. Whereupon it was

*Resolved,* That the thanks of the Association be transmitted to the Mayor for this courtesy.

There being no further business, the Association, on motion, adjourned *sine die.*

---

